# Evaluation of Cryopreservation of Bovine Ovarian Tissue by Analysis of Reactive Species of Oxygen, Toxicity, Morphometry, and Morphology

**DOI:** 10.3390/vetsci11110579

**Published:** 2024-11-19

**Authors:** Camila Bizarro-Silva, Larissa Zamparone Bergamo, Camila Bortoliero Costa, Suellen Miguez González, Deborah Nakayama Yokomizo, Ana Carolina Rossaneis, Waldiceu Aparecido Verri Junior, Mateus José Sudano, Evelyn Rabelo Andrade, Amauri Alcindo Alfieri, Marcelo Marcondes Seneda

**Affiliations:** 1 Laboratory of Animal Reproduction, University of Londrina (UEL), Londrina 86057-970, Brazil; camila.bizarro@pucpr.br (C.B.-S.); larissabergamo1@hotmail.com (L.Z.B.); cbortoliero@gmail.com (C.B.C.); suellenmgonzalez@gmail.com (S.M.G.); deborahyokomizo@gmail.com (D.N.Y.); 2 Department of Pathological Sciences, University of Londrina (UEL), Londrina 86057-970, Brazil; anacrossaneis@gmail.com (A.C.R.); waverri@uel.br (W.A.V.J.); 3 Department of Genetics and Evolution, Federal University of São Carlos, UFSCar, São Carlos 13565-905, Brazil; mjsudano@ufscar.br; 4 Department of Veterinary Medicine, Federal University of Rondônia, UNIR, Rolim de Moura 76940-000, Brazil; evelyn.andrade@unir.br; 5 Laboratory of Animal Virology, University of Londrina (UEL), Londrina 86057-970, Brazil; alfieri@uel.br

**Keywords:** vitrification, DMSO, in vitro culture of preantral follicles, ROS

## Abstract

Ovarian tissue cryopreservation has been widely investigated for preserving female fertility. In the present study, we aimed to compare the effects of three concentrations (1, 1.5, and 3 M) of dimethylsulfoxide (DMSO) on the vitrification of ovarian tissue. The ovarian cortex was divided into control and vitrified groups: (i) 1 M-DMSO, (ii) 1.5 M-DMSO, and (iii) 3 M-DMSO. Follicles from all fragments were analyzed for DMSO-induced deleterious effects, morphological and morphometric aspects, and concentration of reactive oxygen species. Additionally, the fragments were cultured to assess the integrity and return of follicular development post-vitrification. All DMSO concentrations resulted in a higher percentage of degenerated preantral follicles than before the cryopreservation process. After vitrification, the cryopreserved ovarian fragments showed similar percentages of intact follicles; however, the 3 M DMSO concentration differed from the control. Analyzing free radical production, we found that the 3 M DMSO concentration had higher levels of oxidative stress than the lower DMSO. After in vitro cultivation of the vitrified/warmed fragments, the 1 M DMSO concentration exhibited higher percentages of morphologically intact follicles than the other concentrations. Therefore, we suggest that bovine preantral follicles can be cryopreserved in situ with greater efficiency in 1 M DMSO.

## Simple Summary

There is a growing demand for the development of optimal protocols for ovarian tissue cryopreservation in different animal models to enable the maintenance of cell morphology. Therefore, it is crucial to understand the effects of conservation on ovarian cell proliferation and apoptosis to improve follicular development after in vitro culture and/or ovarian tissue transplantation. This study compared the effects of three concentrations (1, 1.5, and 3 M) of dimethyl sulfoxide (DMSO) during vitrification to identify optimal conditions for preserving bovine preantral follicles and to contribute to the improvement of the technique and its future application in assisted reproduction procedures. Following vitrification, cryopreserved ovarian fragments exhibited similar percentages of intact follicles, with only the 3 M DMSO concentration differing from the control. Upon analyzing the free radical production, the 3 M concentration exhibited higher oxidative stress levels than lower DMSO concentrations. Exposure to 3 M DMSO for 30 min led to a higher rate of follicular degeneration. After in vitro cultivation of the vitrified fragments, the 1 M DMSO concentration showed higher percentages of morphologically intact follicles than the other concentrations. Accordingly, bovine preantral follicles could be cryopreserved in situ with greater efficiency in 1 M DMSO.

## 1. Introduction

The cryopreservation of ovarian tissue has been widely explored as a potential strategy to preserve female fertility. Neoplasm therapies, such as radiation and chemotherapy, can lead to premature ovarian failure and infertility owing to the harmful effects of these procedures [1,2]. The potential to preserve fertility prior to treatment can be convenient for patients undergoing such treatments. Over the last few decades, the demand for fertility preservation has dramatically increased [3,4].

In this context, there is a growing need for studies aimed at developing optimal protocols for the cryopreservation of ovarian tissue in different animal models to maintain cell morphology, as well as components of the extracellular matrix [5,6]. Ovarian tissues can be preserved using slow-freezing and vitrification methods. Nevertheless, controversies exist regarding the selection of the most appropriate cryopreservation method [5]. Although slow freezing has been used successfully for several years, this method warrants the use of expensive equipment and leads to intracellular ice crystal formation, which is responsible for changes in the cell morphology and ultrastructure [7,8,9]. However, the vitrification technique, which is a rapid process, does not warrant several resources to be successfully performed and can achieve good results. Moreover, it can vitrify the ovarian tissue and prevent the formation of intracellular crystals, making this technique more attractive [9,10].

Establishing cryopreservation protocols for ovarian tissue in different species presents a considerable challenge owing to the complex architecture of gonadal tissue and cell structures, as well as cryoprotectant-induced toxicity. Glycerol is the agent of choice for most cells because it is generally less toxic than dimethylsulfoxide (DMSO). However, DMSO is more permeable and the typical agent of choice for larger and more complex cells [11]. This characteristic has substantial advantages in the vitrification of both human and mouse embryos when compared to solutions with or without DMSO, and although both protocols were effective, the solution comprising DMSO could elicit superior protective effects under prolonged exposure conditions [12]. However, its greater ability to penetrate the cell membrane can reduce the viability rates of preantral follicles [13]. Preantral follicles are the best alternative to preserve fertility, given the ease of obtaining and isolating the ovarian tissue. These follicles showed optimal performance after freezing and thawing. Nevertheless, additional time is required for the development and growth of competent oocytes [14]. The use of DMSO in the vitrification of preantral follicles may be assisted by increasing solution viscosity to promote vitrification, penetrating cellular membranes to enhance cryoprotection, reducing oxidative stress (OS), and facilitating cellular recovery after thawing [15]. Therefore, comprehensively clarifying the effects of conservation on ovarian cell proliferation and apoptosis in each species, particularly when using bovine models to study females, is pivotal to improving the success of follicular development and gamete production after in vitro culture and/or ovarian tissue transplantation [16].

The objective of the current study was to compare the effects of three concentrations (1, 1.5, and 3 M) of DMSO during vitrification to identify optimal conditions for preserving bovine preantral follicles and to contribute to the improvement of the technique and its imminent application in assisted reproduction procedures.

## 2. Materials and Methods

Unless otherwise specified, all chemicals used in this study were purchased from Sigma-Aldrich (St. Louis, MO, USA). The experiments were conducted in accordance with the Ethics Committee for Animal Experimentation of the State University of Londrina (identification number 061.2022).

### 2.1. Collection and Transport of Ovaries

**Figure 1:**
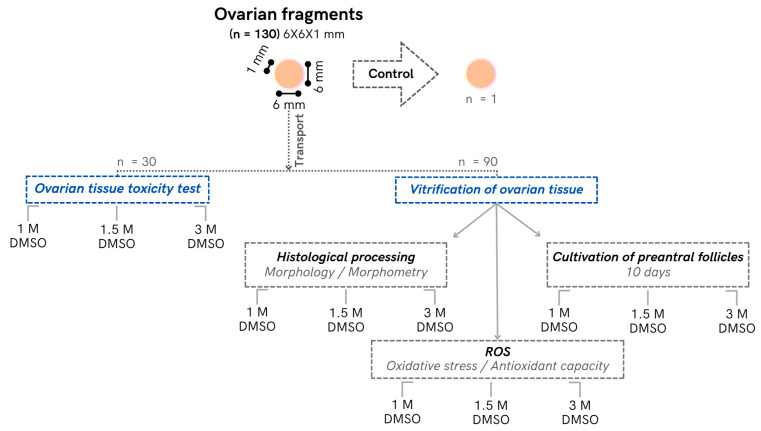
Experimental design of the protocol used for cryopreservation of bovine ovarian tissue.

Pairs of ovaries (*n* = 20) were harvested from a local slaughterhouse from 10 female *Bos indicus* heifers (Nellore breed), aged approximately 24 to 26 months (±1.2 months), showing good body condition (body score 2.5 to 3.5; scale 0 to 5), specifically raised for beef production. After recovery, the ovaries were washed in 70% ethanol for 10 s and sodium chloride saline (JP Farma, São Paulo, Brazil) and then cut into fragments (*n* = 130) of approximately 6 × 6 × 1 mm using a disposable sterile dermatological punch (6 mm; Kolplast, São Paulo, Brazil). One fragment from each ovarian fragment was randomly selected and immediately fixed in Bouin (fresh control); the remaining fragments were transported at a controlled temperature to the laboratory (2 °C; approximately 30 km for 45 min; Figure 1). For fragment transportation, minimum essential medium (MEM; Gibco BRL, Rockville, MD, USA; osmolality 300 mOsm/L, pH 7.2) supplemented with 100 g/mL penicillin and 100 g/mL streptomycin was used.

### 2.2. Evaluation of Deleterious Effects of DMSO on Non-Cryopreserved Ovarian Tissue Fragments (Toxicity Test)

A toxicity test was performed to evaluate the effects of follicle exposure in ovarian tissue fragments to three concentrations of DMSO (1, 1.5, and 3 M), without cryopreservation, on the percentage of degenerated preantral follicles (adapted by Wiltshire et al. [17], Lucci et al. [18]). Ovarian fragments (*n* = 30) were exposed to 1.8 mL of MEM containing 1, 1.5, or 3 M DMSO for 20 min at 20 °C (equilibrium period). After equilibration, cryoprotectants were immediately removed from the tissues. The fragments were washed in MEM at room temperature, fixed in Bouin’s solution for 24 h, and processed for histological analysis. Each concentration was tested using ovarian fragments from ten animals.

### 2.3. Vitrification of Ovarian Tissue

The cryopreservation process was adapted from the experimental protocol described by Praxedes et al. [19]. As previously described, the ovaries were fragmented using a disposable sterile dermatological punch (6 mm^3^), and the cortex of the ovary was fragmented (*n* = 90). For vitrification, the fragments were placed in 2.0 mL cryovials and placed in an equilibration solution (ES; step 1) under conditions similar to the assessment of the harmful effect of DMSO, followed by transfer to the vitrification solution (VS; step 2).

In brief, the equilibrium solution consisted of different concentrations of DMSO (1, 1.5, and 3 M) in MEM. Under the same conditions, the vitrification solution comprised 1 M, 1.5 M, and 3 M of DMSO in MEM, supplemented with 0.25 M sucrose and 10% fetal bovine serum (FBS). The ovarian fragments were exposed to 1.8 mL of ES for 15 min at 4 °C, and after equilibration, the fragments were transferred to 1.8 mL of VS for 15 min at 4 °C. The fragments were then dried (using sterile gauze) and placed in contact with a metal cube surface partially immersed in liquid nitrogen (N_2_) for vitrification for 30 s. Once vitrified, the sample was stored in Liquid N2 (−196 °C) in cryogenic tubes for one week (7 days).

### 2.4. Warming

After 7 days, the vitrified samples were heated. Initially, the cryogenic tubes were exposed to air for 30 s at room temperature and then immersed in a water bath at 39 °C for 3 min. For cryoprotectant removal, the fragments were subjected to three consecutive washes for 5 min in MEM supplemented with 10% FBS and decreasing sucrose concentrations (0.50, 0.25, and 0 M). Finally, the ovary samples were allocated for histological and morphometric analyses, in vitro culture, and response to OS.

### 2.5. Cultivation of Preantral Follicles After Warming

After warming, the vitrified fragments were individually grown on agarose gel support in 1 mL aliquots of culture medium in 24-well culture plates in an incubator at 38.5 °C under an atmosphere of 5% CO_2_ in air and saturated humidity for 10 days. The control culture medium was MEM (Sigma M7278, Spruce Street, St. Louis, MO, USA; osmolality 300 mOsm/L, pH 7.2) supplemented (MEM+) with ITS (insulin 6.25 μg/mL, transferrin 6.25 μg/mL, and selenium 6.25 μg/mL), 0.23 mM pyruvate, 2 mM glutamine, 2 mM hypoxanthine, 1.25 mg/mL of bovine serum albumin (BSA; Gibco BRL, Rockville, MD, USA), 20 UI/mL penicillin and 200 mg/mL of streptomycin; this medium was called MEM+. For preparing the agarose gel, the solution used for gel formation was prepared by dissolving agarose (Agarose Molecular Biology Grade; Kasvi, Brazil) in sterile deionized water (Milli-Q) to obtain a 1.5% solution of agarose (*w*/*v*). After obtaining the gel, 1 cm^3^ supports were prepared [20]. The gel was completely replaced on the sixth day of cultivation. At two-day intervals, the culture medium was replaced with fresh medium at a controlled temperature (38.5 °C). The cultivation period used was based on previous reports on the activation of primordial follicles in bovine species [21].

### 2.6. Histological Processing

For histological analysis of ovarian follicle morphology, fragments of the following groups were fixed by immersion in Bouin for 24 h and maintained in 70% alcohol: control (not vitrified); vitrified/warming (1, 1.5, and 3 M DMSO); and cultured in vitro after cryopreservation. After fixation, the tissues were dehydrated in a graded series of increasing ethanol solutions, cleared in xylene, embedded in paraffin, and then embedded in paraffin blocks for histological analysis. Subsequently, each piece was sectioned at 5 µm with a 5-section interval of the tissue in a rotating microtome (Leica^®^, Wetzlar, Germany) for slide assembly for microscopy. The slides were stained with periodic acid–Schiff (PAS) and hematoxylin. For PAS and hematoxylin staining, the tissue slides were initially dewaxed with xylene and then rehydrated using 70% ethanol before staining with periodic acid for 5 min and Schiff’s reagent for 10 min and counterstained with hematoxylin.

#### 2.6.1. Analysis of Follicular Growth, Morphology, and Degeneration

All sections were examined using optical microscopy (10× and 40×; Nikon, Tokyo, Japan). The preantral follicles were classified according to the following morphological criteria: (1) integrity of the granulosa cells and oocytes, (2) organization of granulosa cells, (3) ooplasmic retraction, (4) presence or absence of pycnotic bodies, and (5) presence of an undulated basement membrane that firmly adhered to the granulosa cells. Based on these criteria, the follicles were considered morphologically intact when the oocyte was morphologically intact with a non-pycnotic nucleus and surrounded by granulosa cells organized in discrete layers, or degenerate when the oocyte was shrunk with a pyknotic nucleus and disorganized, surrounded by granulosa cells isolated from the basal membrane [22]. Follicular development can be classified as (i) primordial (containing a layer of somatic cells known as granulosa cells, flat or flat around the oocyte), (ii) primary (a single layer of cuboid granulosa cells around the oocyte), or (iii) secondary (two or more layers of cubic granular cells [23]). To avoid double counting, the preantral follicles were counted only in the section where the oocyte nucleus was observed. Follicular degeneration was classified as grade 1 when granulosa cells and/or oocyte cytoplasm were damaged, grade 2 when oocytes showed degeneration, and grade 3 when both structures were degenerated [6].

#### 2.6.2. Morphometry of Preantral Follicles Cultured In Vitro

Preantral follicles and captured images were examined to analyze the diameters of follicles and vitrified oocytes. A total of 150 preantral follicles were evaluated in the vitrified treatments with 1 M DMSO (*n* = 50), 1.5 M DMSO (*n* = 50), and 3 M DMSO (*n* = 50). The measurement of follicles and oocytes was performed as described by Silva-Buttkus et al. [18], and the oocyte and follicular diameters were calculated from the arithmetic mean of two perpendicular measurements using an optical microscope OPT HD 0400s (São Paulo, Brazil).

### 2.7. Redox Status

The redox status of non-cultured ovarian tissue samples and samples cultured in vitro for 10 days was determined using colorimetric assays to assess lipid peroxidation, superoxide anion production, and antioxidant capacity, as detailed below. The bovine ovarian tissue samples cultured using different methods were collected and maintained at −80 °C for at least 48 h before processing for the aforementioned assays.

#### 2.7.1. Antioxidant Capacity

The total antioxidant capacity of samples was determined based on the ferric reducing antioxidant power (FRAP) and the ability to quench the 2,2-azinobis-3-ethylbenzothiazoline 6-sulfonic acid (ABST) radical cation, described and adapted according to Pinho-Ribeiro et al. [24]. FRAP and ABTS assays were performed as described by Katalinic et al. [25] and transferred to a 96-well microplate format (Pinho-Ribeiro et al. [24]). Ovarian tissue samples were fragmented and homogenized in 1.15% (500 μL) ice-cold KCl buffer, centrifuged (200× *g*, 10 min, and 4 °C), and the supernatant was collected for assays (adapted by Katalinic et al. [25]). For the FRAP assay, the supernatants (20 μL) were mixed with the freshly prepared FRAP reagent (150 μL), and the reaction mixture was incubated at 37 °C for 30 min. The absorbance was measured at 595 nm (Multiskan GO, Thermo Scientific, Vantaa, Finland). For the ABTS assay, 10 µL of the supernatant was mixed with diluted ABTS solution (200 μL) as described in Hohmann et al. [26]. After 6 min of incubation (25 °C), the absorbance was measured at 730 nm. The results were normalized against a standard Trolox curve (0.02–20 nmol) and expressed as Trolox equivalents per mg of protein.

#### 2.7.2. Lipid Peroxidation

Lipid peroxidation was assessed by measuring thiobarbituric acid reactive substance (TBARS). Ovarian tissue samples were fragmented and homogenized in 1.15% (500 μL) cold KCl buffer, and the homogenate was used to perform the assay (adapted by Hohmann et al. [26]). The homogenates (50 µL) were transferred into microtubes, and FeCl_3_ 1 M (5 µL), ascorbic acid (5 µL), 2.8% TCA (50 µL), and 1% TBA (50 µL) were added, and incubated at 100 °C for 15 min. The supernatants were transferred into a 96-well microplate, and malondialdehyde (MDA), an intermediate product of lipid peroxidation, was determined based on the difference in absorbance at 535 and 572 nm (Multiskan GO, Thermo Scientific). The results were reported as an optical density (OD) of 535–572 nm per mg of protein.

#### 2.7.3. Superoxide Anion Production

The production of superoxide anions was determined based on the reduction of the redox dye nitroblue tetrazolium (NBT). Tissue homogenates in 1.15% KCl buffer (500 µL) were transferred to a 96-well plate, followed by the addition of 100 µL of NBT solution (1 mg/mL) and incubation for 1 h at 37 °C. The supernatant was carefully removed, and the precipitated formazan was solubilized in 120 µL of KOH and 120 µL of DMSO. The OD was measured at 600 nm (Multiskan GO, Thermo Scientific); the results are presented as OD per mg of protein.

### 2.8. Statistical Analysis

The data were initially subjected to tests for normality of residues (Shapiro–Wilk) and homogeneity of variance (Bartlett). To analyze the harmful effects of DMSO on degenerated follicles and the degree of degeneration, data were subjected to ANOVA and Tukey’s tests. The morphology and morphometry of vitrified follicles were subjected to Kruskal–Wallis analysis. For the oxidative stress tests, data were subjected to ANOVA, followed by Tukey’s test. OS analysis was performed using GraphPad Prism 7.0 (GraphPad Software, Inc., La Jolla, CA, USA). All other analyses were performed using the Action 3.1 software version of R 3.0.2 (Campinas, Brazil). Data values were considered statistically significant at *p* < 0.05.

## 3. Results

### 3.1. Morphology Analysis of Degenerated Preantral Follicles After Evaluation of the Harmful Effect of DMSO

**Figure 2:**
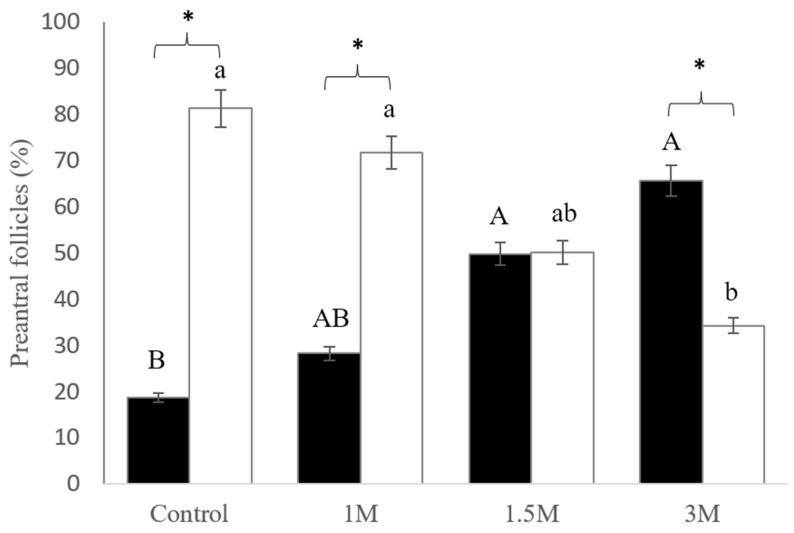
Degenerated (black bars) and intact (white bars) preantral follicles (%) in non-vitrified ovarian fragments (control) and fragments exposed to different DMSO concentrations (1, 1.5, and 3 M) to analyze the deleterious effect of DMSO histologically. Uppercase letters (A, B) indicate significant differences between treatments within the degenerated preantral follicles (*p* < 0.05). Lowercase letters (a, b) indicate significant differences between treatments within intact preantral follicles (*p* < 0.05). (*) indicates significant differences between degenerated and intact follicles within the same treatment group (*p* < 0.05). DMSO, dimethylsulfoxide.

Histological analysis of the ovarian tissue revealed the presence of all follicular categories, with a predominance of primordial follicles, along with intact and degenerate follicles in fragments analyzed for the deleterious effects of DMSO. The percentage of degenerated preantral follicles in the control was 18.7% (80/428). In fragments exposed to 1, 1.5, and 3 M DMSO, the percentages of degenerated preantral follicles were 28.3% (210/741), 49.8% (252/506), and 65.7% (349/531), respectively (Figure 2).

**Figure 3:**
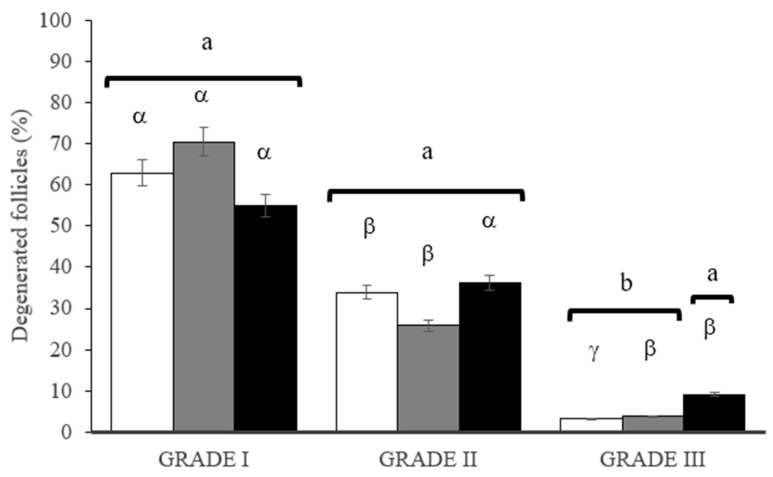
Percentage of grade 1, 2, and 3 degenerated bovine preantral follicles upon exposure to DMSO concentrations of 1 M (white bars), 1.5 M (gray bars), and 3 M (black bars) to analyze the deleterious effect of DMSO. Values followed by lowercase letters (α, β, and γ) indicate significant differences between DMSO concentrations within the same degree of degeneration (*p* < 0.05). Values followed by lowercase letters (a, b) indicate significant differences between the degrees of degeneration (*p* < 0.05). DMSO, dimethylsulfoxide.

Three degrees of degeneration were observed: grade 1, in which granulosa cells and/or oocyte cytoplasm were damaged; grade 2, in which oocytes showed signs of degeneration; and grade 3, in which both structures were degenerated. The percentages of degenerated preantral follicles of grades 1, 2, and 3 in the evaluation of the deleterious effects of DMSO are shown in Figure 3.

A greater proportion of degenerated follicles of grade 1 than grades 2 and 3 was observed in fragments vitrified with 1, 1.5, and 3 M DMSO (*p* < 0.05). Upon analyzing the harmful effects of DMSO, there was no difference between degenerated follicles of grades 1 and 2 when cryoprotectants were used at 1, 1.5, and 3 M DMSO (*p* > 0.05). However, a higher percentage of grade 3 degenerate follicles was observed following exposure of fragments to 3 M DMSO (*p* < 0.05).

Assessing cryoprotectant concentrations, treatment with 1 and 1.5 M of DMSO resulted in higher percentages of grade 1 follicles than grade 2 and 3 follicles (*p* < 0.05). However, treatment with 3 M DMSO resulted in a similar percentage of grades 1 and 2 degenerated follicles (*p* > 0.05), which differed for grade 3 (*p* < 0.05).

### 3.2. Analysis of Follicular Morphology and Integrity of Vitrified Preantral Follicles

**Figure 4:**
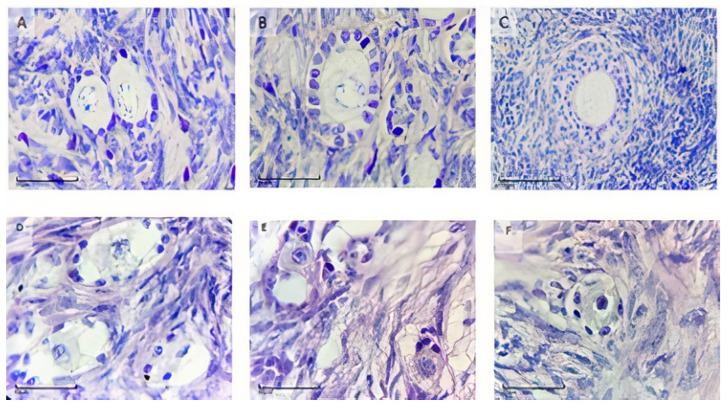
Representative histological images of the morphology of vitrified preantral follicles. (**A**): intact primordial follicle; (**B**): intact primary follicle; (**C**): intact secondary follicle; (**D**): degenerated primordial follicles (cytoplasmic vacuoles); (**E**): degenerated primordial follicles (granulosa cell disorganization); (**F**): degenerated primordial follicle (nuclear retraction). Periodic Acid–Schiff (PAS) and hematoxylin, 100×.

A total of 1842 preantral follicles were evaluated, of which 1086 were intact and 756 were degenerated (Figure 4). Owing to the small number of follicles in the primary and secondary development classes, they were combined and referred to as developing follicles for descriptive statistical analysis.

**Figure 5:**
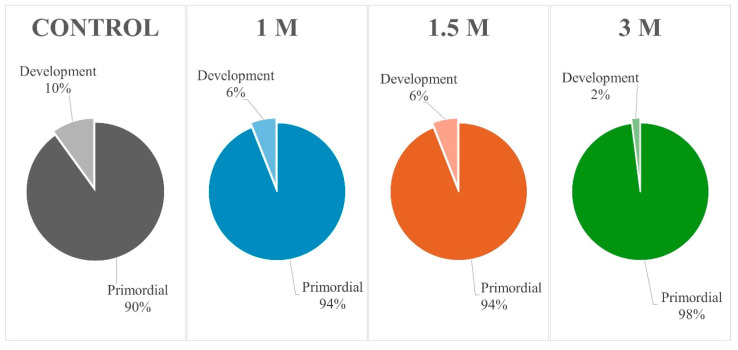
Percentage of primordial and developing follicles in fresh ovarian tissue (control) and vitrification/warming with different DMSO concentrations (1, 1.5, and 3 M). DMSO, dimethylsulfoxide.

In general, after the vitrification process, the proportion of primordial follicles was maintained between the different treatments; therefore, the percentage of this follicular class approached 90–95% (Figure 5). Upon combining data from all treatments, the percentage of intact preantral follicles was 81.3% in fresh ovarian fragments (control), 57.6% in fragments exposed to different concentrations of DMSO at 20 °C and 51.2% in the cryopreserved fragments, respectively.

**Figure 6:**
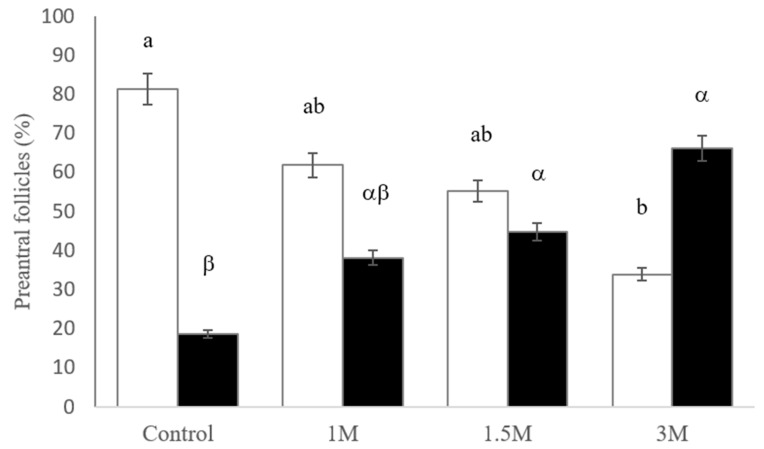
Percentage of intact (white bars) and degenerated (black bars) follicles in fresh ovarian tissue (control, day 0) and after vitrification with different concentrations of dimethyl sulfoxide (DMSO). Values followed by lowercase letters (a, b) indicate significant differences between DMSO concentrations in intact preantral follicles (*p* < 0.05). Values followed by lowercase letters (α, β) indicate significant differences between DMSO concentrations in degenerated preantral follicles (*p* < 0.05).

The percentages of morphologically intact and degenerated preantral follicles (primordial and developing) are shown in Figure 6. After vitrification/warming, ovarian fragments cryopreserved in 1, 1.5, and 3 M of DMSO exhibited similar percentages of intact follicles (*p* > 0.05); however, the percentage of intact follicles with 3 M DMSO differed from the control (81.3% vs. 33.9%, *p* < 0.05). Thus, the 3 M DMSO concentration was significantly more toxic than the 1 and 1.5 M DMSO concentrations in the preantral follicles (66.1%, *p* < 0.05).

After cryopreservation, the differences in the percentage of degenerated follicles between DMSO concentrations (1, 1.5, and 3 M) were non-significant. The percentage of degenerated follicles did not differ significantly between the 1 M DMSO concentration and fresh tissue (*p* < 0.05).

### 3.3. Morphometry of Vitrified Preantral Follicles

**Figure 7:**
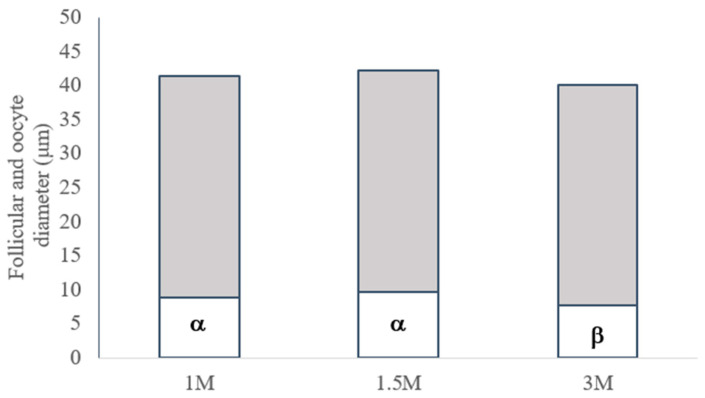
Average follicular (gray section) and oocyte (white section) diameter (μm) of bovine preantral follicles in tissue samples vitrified with different concentrations of dimethyl sulfoxide (DMSO; 1, 1.5, or 3 M). Values followed by different letters (α, β) indicate significant differences in oocyte diameter between DMSO concentrations (*p* < 0.05).

For the morphometric analysis of vitrified follicles, the dimensions of follicles and oocytes were measured in the ovarian tissue vitrified with different DMSO concentrations. The average diameters of follicles and oocytes are shown in Figure 7.

After cryopreservation, the mean diameters of preantral follicles were similar among the three groups vitrified with DMSO (1 M: 32.5 ± 8.9 μm, 1.5 M: 32.6 ± 6.1 μm; and 3 M: 32.3 ± 8.0 μm; *p* > 0.05). Considering the diameter of oocytes present in the follicles, no significant difference was detected between 1 M and 1.5 M DMSO (8.9 ± 2.4 and 9.7 ± 2.1 µm, respectively); however, 3 M DMSO resulted in significantly smaller (7.6 ± 1.7 µm) follicular diameters than the other concentrations (*p* < 0.05).

### 3.4. Analysis of the In Vitro Preantral Follicle Morphology

Upon examining 930 preantral follicles, we identified 465 primordial, 465 developing follicles (primary and secondary), and 56% (522/930) intact follicles. After cultivation, all follicular categories were detected, with a predominance of primary follicles. The percentage of intact cultured preantral follicles is shown in Table 1. The uncultivated control (D0) showed mainly intact follicles (81.3%), ensuring the presence of intact preantral follicles in ovarian samples.

The effects of vitrification/warming with 1, 1.5, and 3 M DMSO on follicles, subsequently cultured in vitro in situ, were analyzed by comparing them with fresh follicles (control). Upon cultivation for 10 days post-vitrification, follicles treated with 1 M DMSO did not differ significantly from fresh follicles (*p* > 0.05). However, the percentage of intact follicles vitrified with 1.5 and 3 M DMSO was similar (*p* > 0.05) and differed from that observed with 1 M DMSO (*p* < 0.05).

**Figure 8:**
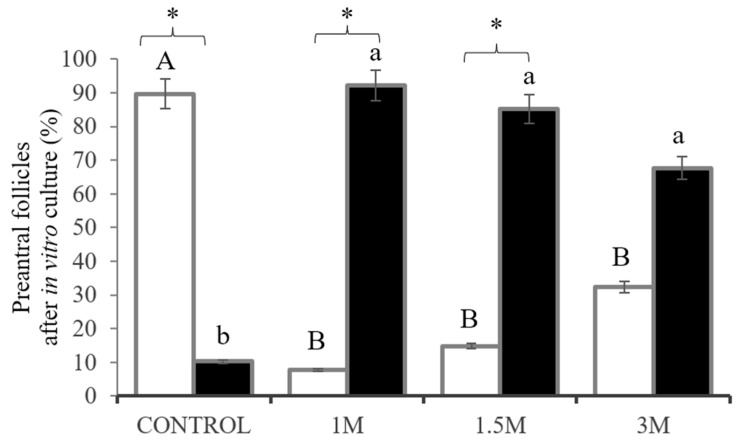
Percentage of preantral follicles in follicular development classified as primordial (white bars) and developing (primary + secondary; black bars) in un-vitrified ovarian tissue (control) and after in vitro cultured ovarian fragments vitrified with different concentrations of DMSO (1, 1.5, and 3 M) for 10 days. Uppercase letters (A, B) indicate significant differences between treatments within primordial follicles (*p* < 0.05). Lowercase letters (a, b) indicate significant differences between treatments in the development (primary and secondary) of follicles (*p* < 0.05). (*) indicates significant differences between primordial and developing follicles within the same treatment group (*p* < 0.05).

Considering the follicular categories, the percentages of primordial and developing follicles are presented in Figure 8. Notably, the percentage of developing follicles in fragments cultured in vitro after vitrification with 1 and 1.5 M of DMSO were similar (*p* > 0.05) when compared with the control. Additionally, treatment with 1 and 1.5 M of DMSO effectively stimulated the activation of primordial follicles and the growth of activated follicles. Furthermore, vitrification with 3 M DMSO could be detrimental to follicular development in vitro.

### 3.5. Redox Status and Antioxidant Capacity

**Figure 9:**
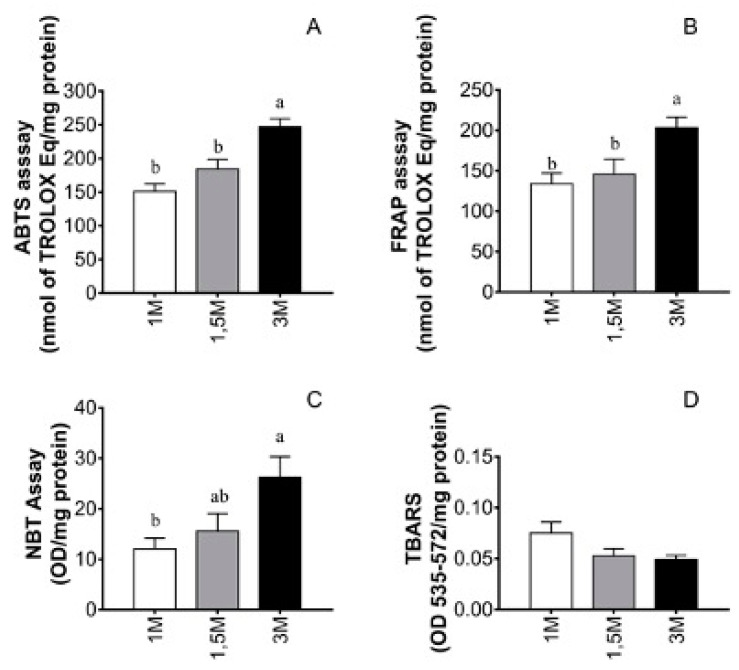
Effects of DMSO concentrations (1 M, 1.5, and 3 M) on oxidative stress (OS) on vitrified ovarian tissue. (**A**) The ability to scavenge free radicals (ABTS assay) and (**B**) the ferric-reducing antioxidant power (FRAP assay) were compared with a Trolox curve. The production of superoxide anion (**C**) and lipid peroxidation (**D**) were determined by the NBT and TBARS tests, respectively. Results are means ± SEM (*n* = 10). One-way ANOVA followed by Tukey’s test. Values followed by lowercase letters (a, b) differ significantly between DMSO concentrations (*p* < 0.05). DMSO, dimethylsulfoxide; NBT, nitroblue tetrazolium; TBARS, thiobarbituric acid reactive substance.

Next, we assessed the oxidative imbalance and found that ovarian tissue vitrified with the highest concentration of DMSO (3 M) exhibited an increased production of reactive oxygen species (ROS) and a decrease in antioxidant capacity when compared with the lower DMSO concentrations (1 and 1.5 M) (*p* < 0.05; Figure 9).

After vitrification/warming, 3 M DMSO, used as a cryoprotective solution, induced a greater amount of free radicals (ABTS; 246.9 ± 11.86) than the other DMSO concentrations examined (1 and 1.5 M: 151.0 ± 11.4 and 184.3 ± 13.9 nmol of TROLOX Eq/mg protein, respectively; *p* < 0.05). Antioxidant assessment based on iron reduction (FRAP) revealed a similar result, with 3 M DMSO exhibiting higher levels (203.5 ± 13.0 nmol of TROLOX Eq/mg protein; *p* < 0.05) than 1 M DMSO (134.1 ± 13.1 nmol of TROLOX Eq/mg protein; *p* > 0.05) and 1.5 M DMSO (145.9 ± 18.5 nmol of TROLOX Eq/mg protein; *p* > 0.05). Considering the colorimetric assay for the superoxide anion (NBT), 3 M DMSO resulted in a greater reduction in NBT (26.2 ± 4.1 OD/mg protein) than the 1 M (12.0 ± 2.1 OD/mg protein; *p* < 0.05). In contrast, the examined DMSO concentrations showed no significant difference in lipid peroxidation (TBARS; *p* > 0.05).

## 4. Discussion

To the best of our knowledge, this is the first study comparing the effects of DMSO at three different concentrations (1, 1.5, and 3 M) as a cryoprotectant in a vitrification protocol for preantral follicles in bovine ovarian tissue. Herein, we evaluated the toxic effects of cryoprotectants on ovarian tissue, follicular integrity, morphological and morphometric aspects, response to OS, and the resumption of follicular development in preantral follicles after cryopreservation. Accordingly, our results revealed that DMSO played an important protective role in the vitrification process at low concentrations (1 M), whereas high concentrations (3 M) could induce toxic effects in ovarian follicles. Therefore, 1 M DMSO may offer greater efficiency in ovarian cryopreservation.

Consistent with the analysis of the harmful effects of DMSO, the morphological changes due to atresia caused by exposure to DMSO were detected mainly in the granulosa cells and/or in the cytoplasm of oocytes. In contrast, previous reports have shown that changes in preantral follicles begin with the retraction of nuclear chromatin and oocyte fragmentation [27]. The tested concentrations of 1.5 and 3 M DMSO could exert a detrimental effect on preantral follicles. Both concentrations showed a low percentage of healthy preantral follicles in bovine ovarian fragments, which may be attributed to cell damage caused by chemical toxicity or osmotic stress, especially at high concentrations when the protective potential of the cryoprotectant is reduced [6,28]. Thus, the interaction of cryoprotectants with tissues in vivo or with biomolecules can exert toxic effects at inadequate concentrations [3].

In the examined follicles, toxicity appeared to be more pronounced when upon treatment with 3 M DMSO (65.7% of degenerated preantral follicles), as evidenced by the signs of grade 3 atresia. The main characteristics of degeneration in the follicles include pyknosis in oocytes and granulosa cells and/or follicular cytoplasm damage due to the presence of vacuoles or retraction. The toxic damage induced by 3 M DMSO is consistent with that reported previously in an experiment using in situ sheep follicles (54.4% [29]). In our study, treatment with a low concentration of DMSO (1 M) resulted in a lower percentage of degenerated follicles (28.3%), and there was no statistical difference when compared with the control group (18.7%; *p* < 0.05). Similar concentrations of DMSO have been shown to exert a protective effect during the vitrification of human and mouse embryos [12]. These findings suggest that low DMSO concentrations can effectively preserve follicular viability while maintaining protective benefits during cryopreservation.

Previous studies have evaluated the toxic levels of different cryoprotectants during vitrification and have observed that ethylene glycol (EG) is more toxic to bovine preantral follicles than DMSO or propylene glycol [18]. In addition, the same study demonstrated that the percentage of morphologically normal follicles was reduced from 96.9% (control) to 49.2% (deleterious effect of DMSO) and still maintained without significant difference after cryopreservation with DMSO (52.5%) [18]. Furthermore, studies that evaluated the genotoxicity of DMSO have characterized it as a molecule with excellent nontoxic solvent properties, as high concentrations of DMSO did not induce breaks in the DNA strand [10,30]. In contrast, the present study demonstrates a higher rate of follicular degeneration after exposure to DMSO for 30 min at 3 M.

Ovarian tissue vitrification is a new and promising technique for germline storage. Despite the optimization of cryopreservation protocols, further studies are crucial to clarify the vitrification process, owing to the complex structure and different types of cells present in the ovarian tissue [31]. Moreover, cooling and warming processes can cause irreversible damage to cryopreserved ovarian tissues [32]. In this study, after warming, follicular integrity was not preserved at the 3 M concentration. The 1.5 M DMSO concentration also showed a high percentage of degenerated follicles, similar to 3 M DMSO post-vitrification. Morphometric responses showed a reduction in oocyte size after cryopreservation with 3 M DMSO. The average dimensions of cryopreserved follicles treated with different DMSO concentrations were similar. Notably, similar diameter values were reported for (non-cryopreserved) ovarian tissue from the same species (*B. indicus*) after in vitro culture for two days in the presence of follicle-stimulating hormone (32.9 µm) [33].

Currently, there is limited evidence regarding cryogenic tolerance and stress caused by preantral follicles that are isolated or incorporated into the cortical tissue. Vitrification has been shown to enhance OS in various tissues [34,35]. Modifying the type or concentration of cryoprotectant can be explored to minimize this problem. The use of DMSO as a cryoprotectant in gamete and embryo cryopreservation offers benefits but also carries risks [15]. Regarding OS, the highest concentration of DMSO (3 M) was found to be more deleterious than the lower concentrations examined in this study. Vitrification has been shown to enhance OS in various tissues [34,35]. Modifying the type or concentration of cryoprotectant can be explored to minimize this problem. However, different concentrations of DMSO reduced the deleterious effects of OS-induced lipid peroxidation and cryopreservation. Similar findings have been observed upon vitrifying ovarian tissue of bovine fetuses using resveratrol [36].

Vitrification can be combined with in vitro culture after warming to assess the quality of stromal cells and vitrified follicles. In situ cultivation in MEM+ can yield better conditions for the development and preservation of the morphology and integrity of the cultured follicles [20]. Our results confirmed the effectiveness of the culture medium in the development of preantral follicles after cryopreservation with DMSO (1 and 1.5 M). Thus, the post-warming viability rate can be used as an indication of the possible restoration of reproductive function. Accordingly, cryopreservation combined with efficient cultivation is a good model for assessing and identifying possible applications in assisted reproduction.

Thus, cryopreservation of ovarian tissues is the main step toward establishing an ovarian tissue bank or for preserving ovarian tissues of patients scheduled to undergo treatment for gonadotoxic cancer to restore fertility at a future time point. It should be noted that the cryopreservation of ovarian tissue is more beneficial for patients with cancer than for the cryopreservation of oocytes [37] and embryos [38], as it can promote the conservation of a large number of follicles [39].

## 5. Conclusions

In conclusion, we demonstrated that a vitrification protocol using an ovarian tissue concentration of 1 M DMSO as a cryoprotectant can effectively preserve female gametes. Under these conditions, the morphological and morphometric characteristics of the preantral follicles in situ were preserved, with the possible resumption of follicular development after vitrification. However, while DMSO protects against ROS, higher concentrations, such as the tested 3 M DMSO, could exert the opposite effect, promoting oxidative damage. Furthermore, our findings underscore the use of bovine ovaries as a model for issues inherent to infertility, and physiological aspects need to be clarified in future investigations.

## Figures and Tables

**Table 1:** Percentage of preantral follicles, morphologically intact and degenerated uncultured (control), and cultured in vitro after vitrification with different DMSO concentrations (1, 1.5, and 3 M).

Follicles	Control	1 M	1.5 M	3 M
Intact	81.3% ^a^ (348/428)	47.7% ^a^ (124/260)	35.7% ^b^ (41/115)	7.1% ^b^ (9/127)
Degenerated	18.7% ^β^ (80/428)	52.3% ^β^ (136/260)	64.3% ^α^ (74/115)	92.9% ^α^ (118/127)

Values followed by lowercase letters (^a, b^|^α, β^) differ significantly (*p* < 0.05).

## Data Availability

Data is contained within the article.
